# ﻿Two new species of *Collybiopsis* (Agaricales, Omphalotaceae) from Eastern North America

**DOI:** 10.3897/mycokeys.107.122634

**Published:** 2024-07-22

**Authors:** Ronald H. Petersen, Karen W. Hughes

**Affiliations:** 1 Ecology & Evolutionary Biology, University of Tennessee, Knoxville, TN 37996-1100, USA University of Tennessee Knoxville United States of America

**Keywords:** *Gymnopus*, Marasmiaceae, new species, Omphalotaceae, phylogeny, taxonomy

## Abstract

Two small gymnopoid fungi from the southern Appalachian Mountains and Massachusetts, *Collybiopsiscomplicata***sp. nov.** and *C.prolapsis***sp. nov.**, are identified and described. A new generic nrITS-LSU phylogeny of *Collybiopsis* places *C.complicata* and *C.prolapsis* in a small clade together with *C.minor*, and an unknown taxon from Arkansas. This clade adds to the growing circumscription of *Collybiopsis* (= *Marasmiellus*).

## ﻿Introduction

Although the southern Appalachian Mountains of the Eastern United States have been explored by numerous mycologists for the last century, not all agarics have been recorded or described. This is especially true of the Great Smoky Mountains National Park (GSMNP) and adjacent regions where L. R. Hesler, together with visiting workers, collected for much of the 20^th^ century, and where the senior author and additional visitors have collected and described fungi for the past 50 years or more (Hesler 1959; Petersen 1977; Desjardin 1989; Mata et al. 2007). Nonetheless, smaller agarics from this species-rich region often remain overlooked. Especially problematic are small litter- and wood-decomposing agarics that dominate the moist understory of the varied conifer-hardwood forests of the southern Appalachian Mountains. Herein, we name and describe two such fungi within *Collybiopsis*, *C.complicata* and *C.prolapsis*, place them within a larger *Collybiopsis* phylogeny, and note that while originally identified from the southern Appalachian area, *C.complicata*, at least, has a wider distribution.

Species within *Collybiopsis* and *Gymnopus* were previously included within *Collybia* s.l., a large polyphyletic genus within the Omphalotaceae consisting of transient basidiomes, convex and often non-striate pilei, variably attached lamellae and robust, non-filiform stipes (compared to *Marasmius*) (Halling 1983; Wilson and Desjardin 2005; Mata et al. 2007). With the advent of molecular methods of assessing fungal relationships, it became clear that morphology alone was insufficient to delineate modern taxonomic relationships and that traditional genera of small saprobic “collybioid” fungi (*Marasmius*, *Marasmiellus* and *Collybia*) were polyphyletic. *Collybia* s.s. was segregated from *Collybia* and remaining taxa were transferred to *Gymnopus* (“gymnopoid fungi”: (Antonín and Noordeloos 1993, 1997). Wilson and Desjardin (2005) examined nrLSU-based phylogenetic relationships among the gymnopoid and marasmioid fungi and designated two unresolved clades, /gymnopus and /marasmiellus. /Marasmiellus contained the type species of *Marasmiellus* and was dominated by members of GymnopussectionVestipedes (stipe surface usually with a vesture, hyphae sometimes diverticulate or coralloid). Mata et al. (2007) used molecular data to examine structure within *Gymnopus*, arriving at clades A-N of “gymnopoid fungi”, confirming placement of the generic type species of *Marasmiellus*, *Marasmiellusjuniperinus*, within *Gymnopus* clade D (Mata et al. 2004). Species within Clade D (/Marasmiellus of Wilson and Desjardin 2005) were transferred to *Marasmiellus* (Oliveira et al. 2019), then *Collybiopsis* (Petersen and Hughes 2021). Other gymnopoid taxa were subsequently segregated from Gymnopus including *Paragymnopus*, *Mycetinis* (Petersen and Hughes 2017), *Paramycetinis* (Petersen and Hughes 2020), and *Pseudomarasmius* (Petersen and Hughes 2020). The most recent treatment of the Omphalotaceae overall which included 191 sequences within the Omphalotaceae showed *Mycetinis* and *Paramycetinis* as basal to *Marasmiellus* and sister to a diverse group of taxa including *Lentinula* and *Rhodocollybia* (Petersen and Hughes 2020). The terms “gymnopoid” and marasmielloid” thus refer to taxa that historically belonged to *Gymnopus* or *Marasmiellus* (spore print white, stipe central, without volva or annulus; lamellae variously attached) but marasmielloid fungi tend to have pale pilei, prostrate and diverticulate hyphae and cheilocystidia arising from horizontal hyphae in the hymenium. Ultimately, both gymnopoid and marasmielloid fungi are defined molecularly as belonging to *Gymnopus* or *Marasmiellus*.

## ﻿Materials and methods

### ﻿Macromorphology

Below, colors of basidiomatal structures within quotation marks (“”) are from Ridgway (1912) and matching colors from Kornerup and Wanscher (1967) are cited alphanumerically by plate, column, and row (i.e. “28E5”).

### ﻿Micromorphology

Observations of microscopic structures were made with an Olympus BX60 research microscope fitted with phase contrast microscopy (PhC). Photos were produced using an Olympus Q-color 5 camera/computer attachment. All micromorphology was accomplished with squash mounts of minute amounts of basidioma tissue in 3% aqueous KOH; in some cases, enough material existed to make a second mount in Melzer’s reagent (cited as IKI) to test for amyloidity.

### ﻿Molecular procedures

DNA was extracted from either dried herbarium specimens or from cultures grown in PD Broth (24g/l Difco Potato Dextrose: Thermo Fisher Scientific, Waltham, Massachusetts) using an E.N.Z.A HP Fungal DNA kit (Omega Bio-Tek Inc., Norcross, GA). The nrITS (Schoch et al. 2012) and nrLSU regions were PCR-amplified using procedures outlined in Hughes et al. (2020b). Primers ITS1F, ITS4, ITS2, and ITS3 were used in various combinations to amplify the whole nrITS region or fragments of the region (White et al. 1989; Gardes and Bruns 1996). Primers LR0R and LR5 were used to amplify the 5’ end of the nrLSU region (Cubeta et al. 1991). PCR products were confirmed by gel electrophoresis. Five µL of the PCR product were treated with 2ul ExoSAP-IT (Thermo-Fisher Scientific) using the manufacturer’s directions. Sanger dideoxy sequencing reactions were performed using BigDye Terminator 3.1 (Thermo-Fisher Scientific) following manufacturers’ directions but with cycles increased to 35. Sanger sequencing was performed by the University of Tennessee UT Genomics Core, College of Arts and Sciences.

**Figure 1.**
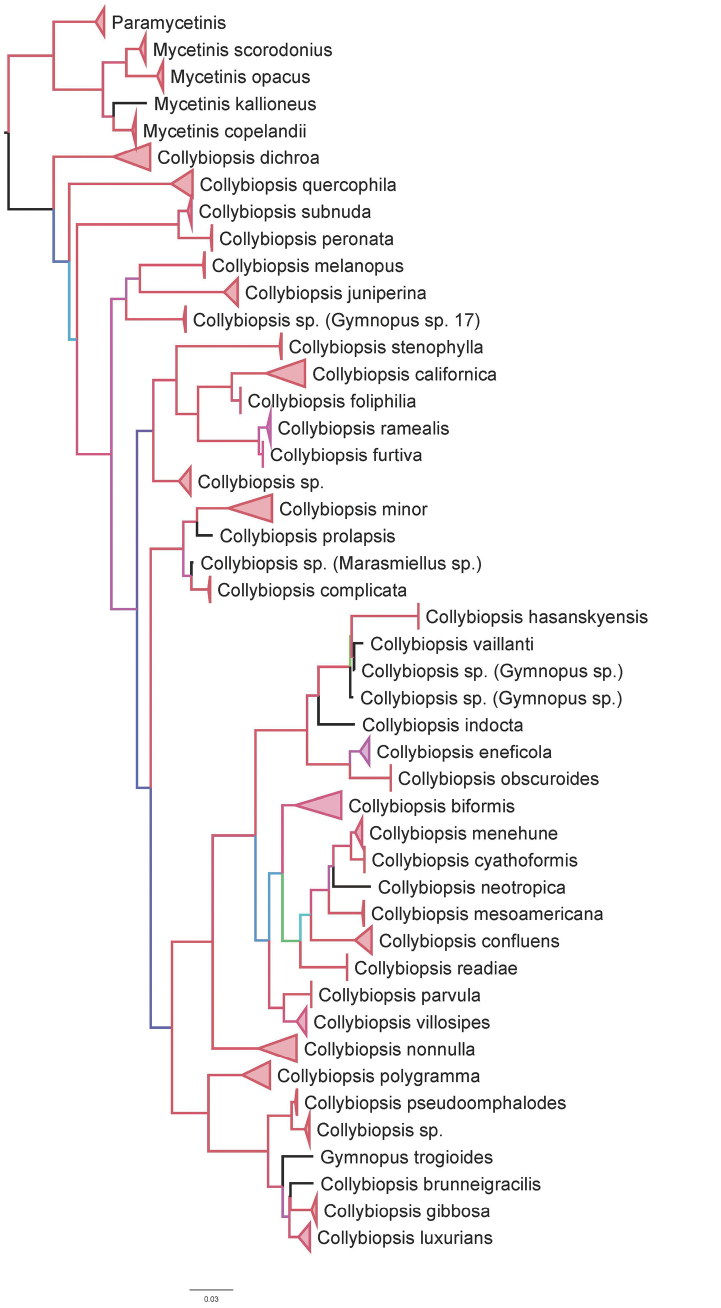
nrITS-nrLSU based Maximum Likelihood consensus tree with 1000 bootstrap replicates. The tree was generated using the web version of IQTree tree (http://iqtree.cibiv.univie.ac.at/) using the best-fit model of evolution (GTR+F+I+G4, AIC criteria). Colors represent branch bootstrap support. Red = 95–100% bootstrap support, Purple = 90–94.9% bootstrap support, Blue = 80–89.9% bootstrap support, Aqua = 70–79.9% bootstrap support and Green = 60–69.9 bootstrap support.

NrITS and nrLSU sequences (Table 1) were concatenated in Geneious R11.1.5 (Geneious 2017) and aligned manually in Aliview (Larsson 2014). The Alignment for *Collybiopsis* had 177 sequences with 1875 columns, 905 distinct patterns, 605 parsimony-informative sites, and 1172 constant sites. The model of evolution was estimated using Model Finder (Kalyaanamoorthy et al. 2017) in W-IQ Tree (Trifinopoulos et al. 2016) as GTR+F+I+G4. This model was implemented in the generation of a Fast ML tree with 1000 bootstrap replicates, using the web version of IQTree tree (http://iqtree.cibiv.univie.ac.at/) (Fig. 1).

**Table 1.** Collections used in phylogenetic analyses.

Name	Location1	Isolate	Voucher	ITS GenBank Identifier	LSU GenBank Identifier	Figure
Collybiopsisaff.villosipes	Australia: Perth	N.L.Bougher NLB470	PERTH:8872252	MT537088	MT537088	Fig. 1
*Collybiopsisbiforma*	USA: TN	TFB13814	TENN-F-065189	KJ416249	KJ189569	Fig. 1
*Collybiopsisbiforma*	USA: NC	TFB13890	TENN-F-065586	KJ416248	KJ189570	Fig. 1
*Collybiopsisbiforma*	USA: TN GSMNP	TFB14250	TENN-F-068108	KJ416246	KJ189568	Fig. 1
*Collybiopsisbiforma*	USA: TN, GSMNP	TFB14251	TENN-F-068109	KJ416245	KJ189567	Fig. 1
*Collybiopsisbrunneigracilis*	Indonesia: Java	AWW01	AWW01-SFSU	AY639412	no	Figs 1, 2
*Collybiopsiscalifornica*	USA: CA	DED8372	SFSU-F-024526	MN413337	no	Fig. 1
*Collybiopsiscalifornica*	USA: OR	iNAT-143113059	no	OQ781003	no	Fig. 1
*Collybiopsiscalifornica*	USA: WA	iNat-29416590	no	OK346494	no	Fig. 1
*Collybiopsiscalifornica*	USA: CA	none	SFSU Wright2941	MN413335	no	Fig. 1
*Collybiopsiscalifornica*	USA: CA	none	SFSU Wright 866	MN413336	no	Fig. 1
*Collybiopsiscalifornica*	Canada: BC	TFB05787	TENN-F-052617	MN413338	no	Fig. 1
*Collybiopsiscomplicata*	USA: Tennessee, GSMNP	TFB09168	TENN-F-055766	DQ450029	no	Figs 1, 2
*Collybiopsiscomplicata*	USA: North Carolina, Macon Co.	TFB13916	TENN-F-065811	OR500517	OR500517	Figs 1, 2
*Collybiopsiscomplicata* as *Marasmiellus* sp.	USA: MA, World’s End, Boston Harbor Islands	HUH-F-00964493	FH:BHI-F447	MF161269	no	Figs 1, 2
*Collybiopsiscomplicata* as *Marasmiellus* sp.	USA: MA, World’s End, Boston Harbor Islands	HUH-F-00964494	FH:BHI-F401	MF161247	no	Figs 1, 2
*Collybiopsiscomplicata* as *Marasmiellus* sp.	USA: MA, World’s End, Boston Harbor Islands	HUH-F-00964495	FH:BHI-F034	MF161165	no	Figs 1, 2
*Collybiopsisconfluens*	USA: NC	TFB14075	TENN-F-067822	KP710281	KP710281	Fig. 1
*Collybiopsisconfluens*	Germany: Thuringia	TFB14114	TENN-F-067864	KP710296	KJ189573	Fig. 1
*Collybiopsisconfluens*	Germany: Thuringia	TFB14115	TENN-F-067865	KP710292	KJ189578	Fig. 1
*Collybiopsisconfluens*	Canada: NB	TFB14389	TENN-F-069053	KP710279	KJ189584	Fig. 1
*Collybiopsisconfluens*	Canada: NB	TFB14409	TENN-F-09073	KP710278	KJ189585	Fig. 1
*Collybiopsisdichroa*	USA: NC	TFB01860	TENN-F-048680	MW396869	MW396869	Fig. 1
*Collybiopsisdichroa*	USA: SC	TFB05459	TENN-F-051775	MW396868	MW396868	Fig. 1
*Collybiopsisdichroa*	USA: NC, GSMNP	TFB09623h1,h2	TENN-F-056584	MW396865-MW396866	MW396865-MW396866	Fig. 1
*Collybiopsisdichroa*	USA:NC	TFB10009h1	TENN-F-056721	KY026654	KY026654	Fig. 1
*Collybiopsisdichroa*	USA: NC	TFB13873	TENN-F-065569	MW396867	MW396867	Fig. 1
*Collybiopsisdichroa*	USA: TN, GSMNP	TFB14111ss1	TENN-F-067859	KY026696	KY026696	Fig. 1
*Collybiopsisdichroa*	USA: TN, GSMNP	TFB14111ss2	TENN-F-067859	KY026697	KY026697	Fig. 1
*Collybiopsisdisjuncta*	USA: MS	TFB14281	TENN-F-068136	KY019643	KY019643	Fig. 1
*Collybiopsisdisjuncta* Type	USA: CT	TFB14339	TENN-F-069172	KJ416252	PP430330	Fig. 1
*Collybiopsiseneficola*	Canada: Newfoundland	10-09-21AV04	TENN-F-069122	KJ128265	no	Fig. 1
*Collybiopsiseneficola*	USA:AK	NA	MICH:PK6975	KP710270	no	Fig. 1
*Collybiopsiseneficola*	USA:AK	NA	MICH:PK6976	KP710271	no	Fig. 1
*Collybiopsiseneficola* Type	Canada: Newfoundland	09-09-26AV13	TENN-F-069123	NR_137613	NG_059502	Fig. 1
*Collybiopsisfilamentipes* Type	USA: TN	TFB13962	TENN-F-065861	MN897832	MN897832	Fig. 1
*Collybiopsisfilamentipes* Env. Samp.	Canada: Alberta	KTRF390	NA	MG433317	no	Fig. 1
*Collybiopsisfurtiva*	USA: North Carolina, Highlands	DED3973	SFSU-F-024523	MN413339	no	Fig. 1
*Collybiopsisfurtiva* Type	USA:	DED4425	SFSU DED4425	DQ450031	AF042650	Fig. 1
*Collybiopsisfurtiva*	USA: North Carolina, Macon Co.	DED4584	SFSU-F-024508	MN413340	no	Fig. 1
*Collybiopsisfurtiva*	USA: North Carolina, Coweeta	DED5796h1	SFSU-F-024524h1	MN413341	no	Fig. 1
*Collybiopsisfurtiva*	USA: North Carolina, Coweeta	DED5796h2	SFSU-F-024524h2	MN413342	no	Fig. 1
*Collybiopsisfurtiva*	USA: GA	TFB04796	TENN-F-051097	MN413343	MW396879	Fig. 1
*Collybiopsisgibbosa*	Australia: NT	NA	MEL:2382838	KP012713	KP012713	Fig. 1
*Collybiopsisgibbosa*	Brazil: Amapa	NA	URM 90012	KY061202	KY061202	Fig. 1
*Collybiopsisgibbosa*	Brazil: Amapa	NA	URM 90006	KY061203	KY061203	Figs 1, 2
*Collybiopsishasanskyensis* Type	Russia: Far Eastern	TFB11846	TENN-F-060730	MN897829	no	Fig. 1
*Collybiopsishasanskyensis*	Russia: Far Eastern	TFB11847	TENN-F-060731	MN897830	no	Fig. 1
*Collybiopsisindocta*	Argentina	TFB08605	TENN-F-054944	MW396870	MW396870	Fig. 1
*Collybiopsisjuniperina*	Argentina	TFB10782	TENN-F-058988	KY026661	KY026661	
something wrong Collybiopsisvaillantii	USA: TN, GSMNP	TFB13739	TENN-F-065155	KY026676	KY026676	Fig. 1
*Collybiopsisjuniperina*	USA: LA	TFB9889	TENN-F-59540	AY256708	AY256708	Fig. 1
*Collybiopsisluxurians*	Switzerland	TBF04283ss10	TENN-F:050619	KJ416240	PP430331	Figs 1, 2
*Collybiopsisluxurians*	USA: LA	TFB09121	TENN-F-055748	KY026649	KY026649	Figs 1, 2
*Collybiopsisluxurians*	USA: NC	TFB10350	TENN-F:057910	AF505765	not done	Figs 1, 2
*Collybiopsisluxurians*	USA: NC	TFB14060	TENN-F:067806	MW396871	MW396871	Figs 1, 2
*Collybiopsismelanopus* Type	Indonesia: Java	AWW54	SFSU:AWWilson 54	AY263425, NR_137539	AY639422., NG_060624	Fig. 1
*Collybiopsismenehune*		AWW15	SFSU:AWWilson 15	AY263443	AY639424	Fig. 1
*Collybiopsismenehune*	USA: HI	TFB11587	DEH2320	DQ450043	no	Fig. 1
*Collybiopsismenehune*	India		CUH:AM074	KJ778753	no	Fig. 1
*Collybiopsismenehune* Type	Indonesia: Java	DED5866	SFSU: DED5866	AY263426	no	Fig. 1
*Collybiopsismesoamericana*	Costa Rica	REH7379	NYBG REH7379	AF505768	no	Fig. 1
*Collybiopsismesoamericana*	Costa Rica	TFB10411	TENN-F-058106	DQ450036	no	Fig. 1
*Collybiopsisminor*	USA: South Carolina	TFB05434	TENN-F-051792	MW396872	MW396872	Fig. 1
*Collybiopsisminor* Type	USA: TN, GSMNP	TFB11930	TENN-F-067806	MN413334, NG_228867	MW396880	Figs 1, 2
*Collybiopsisneotropica*	Costa Rica	TFB10416	TENN-F-058113	AF505769	no	Fig. 1
*Collybiopsisnonnulla*	USA: MS	TFB14278	TENN-F-068133	KY026701	no	Fig. 1
*Collybiopsisnonnulla*	USA: MS	TFB14492	TENN-F-069193	MW396873	MW396873	Fig. 1
*Collybiopsisnonnulla v. attenuatus*	Cameroon	NA	RAK369.2	MN930621	no	Fig. 1
*Collybiopsisnonnulla v. attenuatus*	Cameroon	NA	RAK372.2	MN930622	no	Fig. 1
*Collybiopsis nonnullus v. attenuatus*	Indonesia: Java	AWW05	SFSU: AWWilson05	AY263445	AY639445	Fig. 1
*Collybiopsis nonnullus v. attenuatus*	Indonesia: Java	AWW55	SFSU: AWWilson55	AY263446	no	Fig. 1
*Mycetinisopacus*	USA: TN	BM888	TENN-F-070567	MW396878	no	Fig. 1
*Mycetinisopacus*	USA: MS	TFB09071	TENN-F-054871	MW396877	MW396877	Fig. 1
*Collybiopsisparvula*	Costa Rica	TFB10422	TENN-F-058116	AF505774	no	Fig. 1
*Collybiopsisparvula*	Mexico	NA	SR83-10MX	KT697977	no	Fig. 1
*Collybiopsisparvula* Type	Costa Rica	TFB10419	TENN-F-058113	NR_119584, DQ450060	no	Fig. 1
*Collybiopsisparvula*	Costa Rica	TFB10421	TENN-F-058115	DQ450061	no	Fig. 1
*Collybiopsisparvula*	Costa Rica	TFB10425	TENN-F-058119	DQ450062	no	Fig. 1
*Collybiopsisperonata*	Belgium, Dinante	TFB13743	TENN-F-065121	KY026677	KY026677	Fig. 1
*Collybiopsisperonata*	USA: GA	TFB14617	TENN-F-069322	KY026738	KY026738	Fig. 1
*Collybiopsisperonata*	Unknown	NA	CBS223.37	MH855896	no	Fig. 1
*Collybiopsispolygramma*	Puerto Rico	TFB09628	TENN-F-056589	DQ450028	no	Figs 1, 2
*Collybiopsispolygramma*	Korea	TFB12806	SFC20120821-64	KJ609162	no	Figs 1, 2
*Collybiopsispolygramma*	Puerto Rico	NA	PR2542TN	AY842954	no	Figs 1, 2
*Collybiopsispolygramma*	India	NA	CUH:AM082	KJ778752	no	Figs 1, 2
*Collybiopsispolygramma*	Brazil: Amapa	NA	URM90015	KY074640	no	Figs 1, 2
*Collybiopsispolygramma*	Brazil: Para	NA	URM90016	KY074641	no	Figs 1, 2
*Collybiopsispolygramma*	Brazil: Para	NA	URM90017	KY074642	no	Figs 1, 2
*Collybiopsispolygramma*	China, Hunan	NA	MHHNU 30912	MK214392	no	Figs 1, 2
*Collybiopsispolygramma*	China, Jiangxi	NA	HFJAU0425	MN258643	no	Figs 1, 2
*Collybiopsispseudoluxurians* holotype	USA: Mississippi	TFB14290	TENN-F-068144	KY026702, NR_137863	KJ416242	Figs 1, 2
*Collybiopsispseudoomphalodes*	Costa Rica	REH7348	NYBG REH7348	AF505762	no	Figs 1, 2
*Collybiopsispseudoomphalodes*	Puerto Rico	NA	PR24TN	AY842957	no	Figs 1, 2
*Collybiopsisperonata*	Russia:	LE-BIN1364	no voucher specimen	KY026755	KY026755	Fig. 1
*Collybiopsisquercophilia*	Slovakia	TFB14570	TENN-F-069267	KY026729	KY026729	Fig. 1
*Collybiopsisquercophilia*	USA: CA	TFB14615	TENN-F-069320	KY026736	KY026736	Fig. 1
*Collybiopsisquercophilia*	USA: CA	TFB14616	TENN-F-069321	KY026737	KY026737	Fig. 1
*Collybiopsisquercophilia*	USA: CA	NA	SFSU:25220	KY026761	KY026761	Fig. 1
*Collybiopsisramealis*	Belgium	BR72_41	BR<BEL>:72-41	MW396875	MW396875	Fig. 1
*Collybiopsisramealis*	UK: Scotland	TFB03772	TENN-F-050509	MN413350	MW396885	Fig. 1
*Collybiopsisramealis*	UK: Scotland	TFB06989	TENN-F-055908	MN413372	MW396883	Fig. 1
*Collybiopsisramealis*	Sweden	TFB13520	TENN-F-062867	JF313670	OR500520	Fig. 1
*Collybiopsisramealis*	Belgium	TFB13759	TENN-F-065136	MN413344	MN413344	Fig. 1
*Collybiopsisramealis*	Belgium	TFB13769	TENN-F-065145	MN413345	MN413345	Fig. 1
*Collybiopsisramealis*	Germany	TFB14140c1	TENN-F-067890	MN413355	OR500518	Fig. 1
*Collybiopsisramealis*	Germany	TFB14150c1	TENN-F-067900c1	MN413363	OR500519	Fig. 1
*Collybiopsisramealis*	Germany	TFB14163h1	TENN-F-067912	MN413351	MN413351	Fig. 1
*Collybiopsisramealis*	Germany	TFB14163h2	TENN-F-067913	MN413352	MN413352	Fig. 1
*Collybiopsisramealis*	Slovakia	TFB14555	TENN-F-069251	MW405779	MW396884	Fig. 1
*Collybiopsisramealis*	Slovakia	TFB14556	TENN-F-069252	MN413369	MN413369	Fig. 1
*Collybiopsisramealis*	Slovakia	TFB14559h1	TENN-F-069255h1	MN413370	PP430332	Fig. 1
*Collybiopsisramealis*	Slovakia	TFB14559h2	TENN-F-069255h2	MN413371	PP430332	Fig. 1
*Collybiopsisreadiae*	New Zealand	TFB07571	TENN-F-053687	DQ450034	no	Fig. 1
*Collybiopsisreadiae*	New Zealand: Buller District	TFB13056	TENN-F-061061	KJ416244	no	Fig. 1
*Collybiopsisreadiae*	New Zealand	NA	PDD: 95844	HQ533036	no	Fig. 1
*Collybiopsis* sp.	Australia: Christmas Island	N.L.Bougher NLB 1292	PERTH:08827494	ON715771	ON715771	Fig. 1
*Collybiopsis* sp. (Gymnopus sp. 17)	USA: CT	TFB14334h1	TENN-F-068185	KY026707	KY026707	Fig. 1
*Collybiopsis* sp. (Gymnopus sp. 17)	USA: CT	TFB14334h2	TENN-F-068185	KY026708	KY026708	Fig. 1
*Collybiopsis* sp. “*prolapsis*”	USA: Georgia	TFB04800	TENN-F-051101	MW396874	MW396874	Figs 1, 2
*Collybiopsis* sp. (*Gymnopus* sp.)	USA: WV	NA	WRW05-1170	KY026764	KY026764	Fig. 1
*Collybiopsis* sp. (VC-2017f)	Brazil: Paraiba	NA	URM 90043	KY321573	KY321573	Figs 1, 2
*Collybiopsis* sp. (VC-2017f)	Brazil: Paraiba	NA	URM 90042	KY321574	KY321574	Figs 1, 2
*Collybiopsis* sp. (VC-2017f)	Brazil: Paraiba	NA	URM 90045	KY321575	KY321575	Figs 1, 2
*Collybiopsis* sp. (VC-2017f)	Brazil: Para	NA	URM 90051	KY321568	KY321568	Fig. 1
*Collybiopsis* sp. (VC-2017f)	Brazil: Para	NA	URM 90053	KY321570	KY321570	Fig. 1
*Collybiopsis* sp. Env. Samp.	USA: California	Environmental Sample	none	DQ273359	no	Fig. 1
*Collybiopsisstenophylla*	USA: North Carolina, Macon Co.	TFB11558	TENN-F-059443	DQ450032	no	Fig. 1
*Collybiopsisstenophylla*	USA: North Carolina, Macon Co.	TFB11559	TENN-F-059444	DQ450033	no	Fig. 1
*Collybiopsisstenophylla*	USA: Georgia	TFB04798	TENN-F-051099	MN413330	MW396879	Fig. 1
*Collybiopsisstenophylla*	Belgium	TFB13770	TENN-F-065146	MN413346	MW396882	Fig. 1
*Collybiopsisstenophylla*	USA: Tennessee, GSMNP	TFB13998	TENN-F-065943	MN413331	MW396886	Fig. 1
*Collybiopsissubcyathiformis*	Brazil: Para	NA	URM90023	KY404982	KY404982	Fig. 1
*Collybiopsissubcyathiformis*	Brazil: Para	NA	URM 90022RNA	KY404983	KY404983	Fig. 1
*Collybiopsissubnuda*	USA: TN, GSMNP	TFB12577	TENN-F-061138	KY026667	FJ750262	Fig. 1
*Collybiopsissubnuda*	USA: NC, Macon Co.	TFB14043	TENN-F-065984	MW396876	MW396876	Fig. 1
*Collybiopsissubnuda*	USA: WV	NA	WRW08-462	KY026765	KY026765	Fig. 1
*Collybiopsistrogioides* Type	Indonesia: Java	AWW51	AWW51-SFSU	NR_152884	NG_228715	Fig. 1
*Collybiopsisvaillantii*	USA: TN, GSMNP	TFB13739	TENN-F-065115	KY026676	KY026676	Fig. 1
*Collybiopsisvelosipes*	USA: CA	TFB09539	TENN-F-056252	DQ450058	no	Fig. 1
*Collybiopsisvillosipes*	New Zealand: Fiordland	TFB12836	TENN-F-060951	KJ416255	FJ750264	Fig. 1
*Collybiopsisvillosipes*	USA: CA	inaturalist.org/observations/2708886	NA	MF163171	no	Fig. 1
*Collybiopsisminor*	USA: South Carolina	TFB06284	TENN-F-052933	MW405778	MW396881	Fig. 1
Environmental Sample	USA: Oregon	clone FON_f09	none	HM488468	no	Fig. 1
Environmental Sample-soil	USA: Oregon	clone FON_h10	none	HM488469	no	Fig. 1
*Gymnopanellanothofagi*	Chile: Aisen	PSL 411	SGO163624	KT906426	KT906426	Fig. 1
*Gymnopanellanothofagi*	Chile: Aisen	PSL 414	SGO163625	KT906425	KT906425	Fig. 1
*Collybiopsisobscuroides*	Sweden: Jamtland	NA	GB-0053811	KX958398	KX958398	Fig. 1
*Collybiopsisobscuroides*	Norway: Svalbard	NA	GB-0150514	KX958399	KX958399	Fig. 1
*Gymnopusperonata*	Canada: BC	NA	UBC F28402	KP454027	no	Fig. 1
*Gymnopus* sp. (VC-2017k)	Brazil: Paraiba	NA	URM 90054	KY404984	KY404978	Fig. 1
Gymnopus sp.	Japan: Okinawa	Ns8-1	none	LC504922	no	Fig. 1
Gymnopus sp. (root sample)	Sweden	olrim406	none	KY352520	no	Fig. 1
*Gymnopus* sp.	USA: MS, Boston Harbor Islands	BHI-F523a	FH:BHI-F523a	MF161290	no	Fig. 1
*Gymnopus* sp. (Not in Collybiopsis paper)	Costa Rica	TFB10494	TENN-F-058602	KY026660	no	Fig. 1
Marasmiaceae sp.	USA: FL	NA	FLAS-F-69007	OP163218	no	Fig. 1
*Marasmiellis* sp.	USA: Arkansas	RA7L5-13a (leaf litter)	none	MK234195	no	Figs 1, 2
*Marasmiellusfoliiphila*	India	none	CUH AM090	KP317637	no	Fig. 1
*Marasmiellusfoliiphila*	India	none	CUH AM101	KP317638	no	Fig. 1
*Marasmiellus* sp.	Mexico: Oaxaca	P196 (soil)	NA	KR135355	no	Fig. 1
*Marasmiellus* sp.	USA:	14147	NA	MW023100	no	Fig. 1
*Mycetiniscopelandii*	USA: CA	TFB08084h1	TENN-F-55408 haplotype h1	KY696750	KY696750	Fig. 1
*Mycetiniscopelandii*	USA: CA	TFB08084h2	TENN-F-55408 haplotype h2	KY696751	KY696751	Fig. 1
*Mycetiniskallioneus*	Norway: Svalvard	NA	GB-0150513	KX958397	KX958397	Fig. 1
*Mycetinisopacus*	USA: MS	TFB14490h1	TENN-F-069200 h1	KY696768	KY696768	Fig. 1
*Mycetinisopacus*	USA: MS	TFB14490h2	TENN-F-069200 h2	KY696769	KY696769	Fig. 1
*Mycetinissalalis*	Canada: BC, Vancouver Island	NA	DAOM:175251	KX752265	KX752265	Fig. 1
*Mycetinisscorodonius*	Sweden	TFB03785	TENN-F-050522	KY696731	KY696731	Fig. 1
*Mycetinisscorodonius*	USA: NC	TFB03071	TENN-F-050689	KY696733	KY696733	Fig. 1
*Mycetinisscorodonius*	USA: TN, GSMNP	TFB03708	TENN-F-050696	KY696734	KY696734	Fig. 1
*Mycetinisscorodonius*	Canada: Nova Scotia	TFB05031	TENN-F-051442	KY696739	KY696739	Fig. 1
*Mycetinisscorodonius*	USA: NY	TFB04969	TENN-F-053466	KY696741	KY696741	Fig. 1
*Mycetinisscorodonius*	Canada: Nova Scotia	TFB05025	TENN-F-053467	KY696742	KY696742	Fig. 1
*Mycetinisscorodonius*	USA: NT	TFB04939	TENN-F-053471	KY696746	KY696746	Fig. 1
*Mycetinisscorodonius*	USA: ME	TFB05005	TENN-F-053474	KY696748	KY696748	Fig. 1
*Paramycetinisaustrobrevipes*	Australia: Tasmania	TFB03585	TENN-F-053181	KY026638	KY026638	Fig. 1
*Paramycetinisaustrobrevipes*	Australia: Tasmania	TFB03591	TENN-F-053146	KY026637	KY026637	Fig. 1
*Paramycetinisaustrobrevipes* Type	Australia: Tasmania	TFB04033	TENN-F-050135	KY026622	KY026622	Fig. 1
*Paramycetiniscaulocystidiatus* Type	New Zealand	TFB07148	TENN-F-054050	KY026645	KY026645	Fig. 1
*Paramycetiniscaulocystidiatus*	New Zealand	TFB07572	TENN-F-053683	KY026642	KY026642	Fig. 1
*Paramycetiniscaulocystidiatus*	New Zealand	TFB07588	TENN-F-053721	KY026643	KY026643	Fig. 1
*Paramycetiniscaulocystidiatus*	New Zealand	TFB07589	TENN-F-053725	KY026644	KY026644	Fig. 1
*Collybiopsismelanopus*	Not Indicated	NA	CUH AM093	KP100305	KP100305	Fig. 1
*Collybiopsismesoamericana* Type	Costa Rica	TFB11005	TENN-F-058613	NR_119583	KY019632	Fig. 1
*Collybiopsisfolliphilia* Type	India	NA	CUH:AM090	NR_154176	NG_060320	Fig. 1

In addition, Bayesian analysis was performed on the *Collybiopsis* alignment in Geneious 11.1.5 using the MrBayes plugin (Huelsenbeck and Ronquist 2001) with a GTR model of evolution (4 Gamma Categories, nst=6, and basefreq=estimated). The MCMC search was carried out with 4 chains for 1,100,000 generations with sampling every 1000 generations. The first 100,000 trees were discarded when likelihood values had reached convergence. Convergence was assessed by ensuring that the average standard deviation of split frequencies was below 0.01. Posterior probabilities were estimated by sampling trees generated after likelihood values had reached equilibrium.

**Figure 2.**
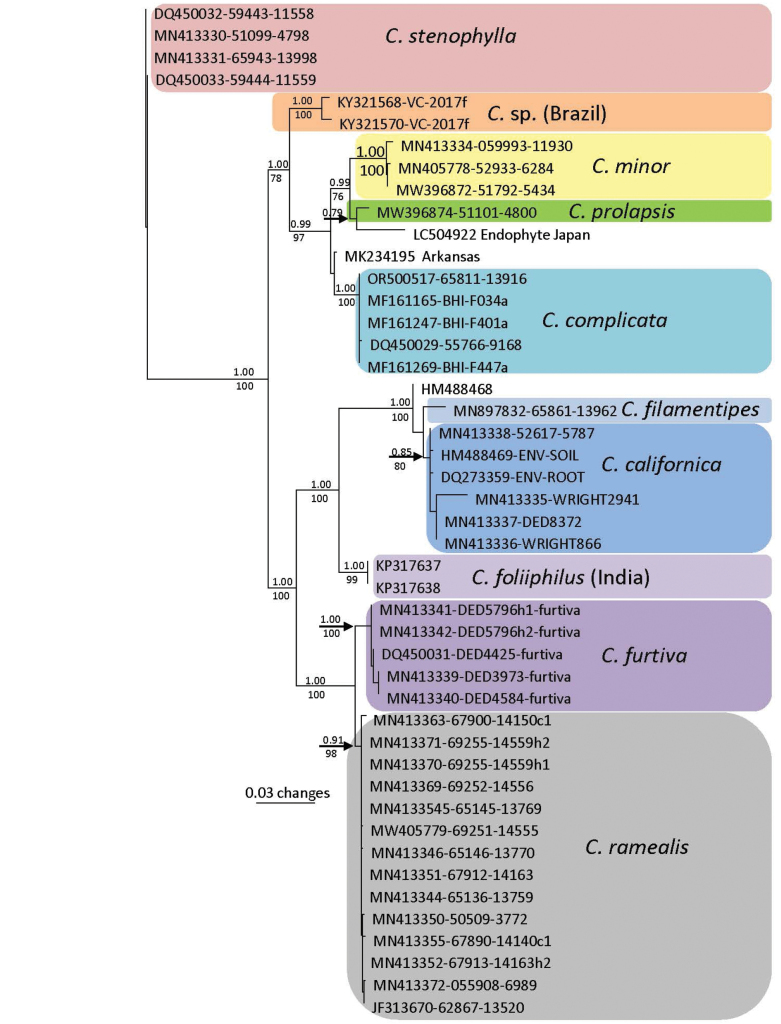
NrITS-nrLSU based Maximum Likelihood consensus tree of the *C.complicata*/*C.prolapsis* clade with 1000 bootstrap replicates. The tree was generated using the web version of IQTree tree (http://iqtree.cibiv.univie.ac.at/) using the best-fit model of evolution (TIM2+F+I+G4, AIC criteria). Included taxa were selected based on the Bayesian analysis which showed the *C.complicata* group and the *C.ramealis* group as sister taxa. Bootstrap support is indicated below the branches and Bayesian Posterior probabilities above the branches.

The alignment for the *C.complicata* subgroup contained 34 sequences with 366 distinct patterns, 182 parsimony-informative sites and 1510 invariant sites. The best-fit model of evolution was estimated using Model Finder in W-IQ-Tree as TIM2+F+I+G4. This model was implemented in the generation of a Fast ML tree with 1000 bootstrap replicates, using the web version of IQTree tree (http://iqtree.cibiv.univie.ac.at/) (Fig. 2). Bayesian analysis was performed as described above.

Taxa used in both analyses are given in Table 1. Collections retained at TENN have both a field number (TFB = Tennessee Field Book) and a TENN accession number (TENN-F-XXXXXX).

## ﻿Results

The phylogenetic position of *C.prolapsis* and *C.complicata* within *Collybiopsis* varies between Maximum Likelihood and Bayesian analyses. In Maximum Likelihood analysis, the *C.prolapsis*/*C.complicata* clade is sister to a large clade containing elements of *Collybia* sects. *Subfumosae*/*Vestipedes* (Fig. 1.). In Bayesian analysis, the *C.prolapsis*/*C.complicata* clade is sister to a clade containing *C.ramealis*. This is the only major difference between the topologies of the two phylogenetic analyses. Other differences include minor differences in the position of Gymnopus sp. 17, an unnamed *Collybiopsis*, and *C.quercophilia*. The nrITS sequences for collections within *C.complicata* (Table 1) are genetically identical with the exception of a 1bp C/T transition in TENN-F-065811 (0.17% difference). In contrast, GenBank accession OR500517 has 6 differing base pairs (1.05%).

The pileipellis structure of *C.prolapsis* and *C.complicata* is similar to that of the infrageneric *Collybia* sects. *Subfumosae* and *Vestipedes* (Halling 1983) on the one hand, and to the cheilocystidial structure of these two species to the *Collybiopsisramealis* group (Petersen and Hughes 2021) on the other. Cheilocystidial morphology also comes close to that of *C.minor*, somewhat morphologically disjunct from the *C.ramealis* group. In all cases, however, molecular sequences clearly separate the *C.ramealis* clade, the *C.complicata*/*prolapsis* clade and the *Collybiopsissubfumosae*/*vestipedes* clade.

### ﻿Taxonomy

#### ﻿ Collybiopsis complicata

Taxon classificationFungiAgaricalesOmphalotaceae

R.H. Petersen sp. nov.

76F509BF-8A8E-5DDE-AF52-078F0AFF1602

Index Fungorum: IF901185

Figs 3 , 4 , 5 , 6 , 7 , 8 , 9 , 10 , 11

##### Holotype.

Tennessee, Blount Co., Great Smoky Mountains National Park, Metcalf’s Bottoms Picnic Area, 9.VI.1997, coll. Ronald H. Petersen, TFB 9168 (TENN-F-055766).

##### Diagnosis.

1) Basidiomata marasmielloid/gymnopoid, gracile, small, with slender stipe; 2) pileus pigmented, especially over disc; 3) pileipellis composed of stalked-coralloid structures and lobed repent hyphae; 4) stipe fully vestured; 5) clamp connections present; 6) cheilocystidia prominent, similar to pileipellis elements; 7) basidiospores 5–8 × 3–4 µm.

##### Etymology.

Cheilocystidia with complex, branched structure; also complex distribution, from southern Appalachians to New England.

##### Description.

**Figure 3.**
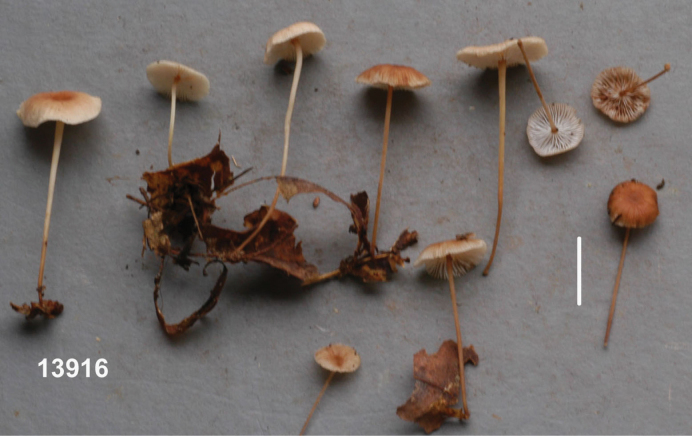
*Collybiopsiscomplicata*. Basidiomata. TFB 13916 (TENN-F-065811). Scale bar: 10 mm.

Basidiomata (Fig. 3) scattered-gregarious >50 basidiomata in <1^2^ m, marasmielloid or miniature collybioid. Pileus 6–14 mm broad, shallowly convex to plane by maturity, matt, delicately rivulose-striate; disc “snuff brown” (5E8), outward “sayal brown” (6C5) to fleshy tan. Lamellae free to adnexed but seceding in drying to become removed from stipe apex, total lamellae 47–64; through lamellae 24–26, in two major ranks with no anastomosis, 1.5–2 mm broad, subventricose to ventricose by maturity, knife-edged, dull pale tan-gray (near “tilleul buff” 7B2), bleaching to off-white, often (but not exclusively) becoming pale cream-colored with necropigment; lamellulae in one rank, less than ½ the length through lamellae. Stipe 7–30(–45) × 0.7–1.2 mm, terete and remaining so upon drying, minutely vestured overall, somewhat darker than lamellae apically, downward fleshy tan to fleshy brown, drying matt; vesture on upper stipe of scattered delicate squamules composed of individual caulocystidia, on lower stipe a solid turf (but never felty), straw-colored to “light ochraceous buff” (5A4); insertion insititious on fine twigs and leaves; superficial litter-binding mycelium not observed. Odor and taste negligible.

**Figure 4.**
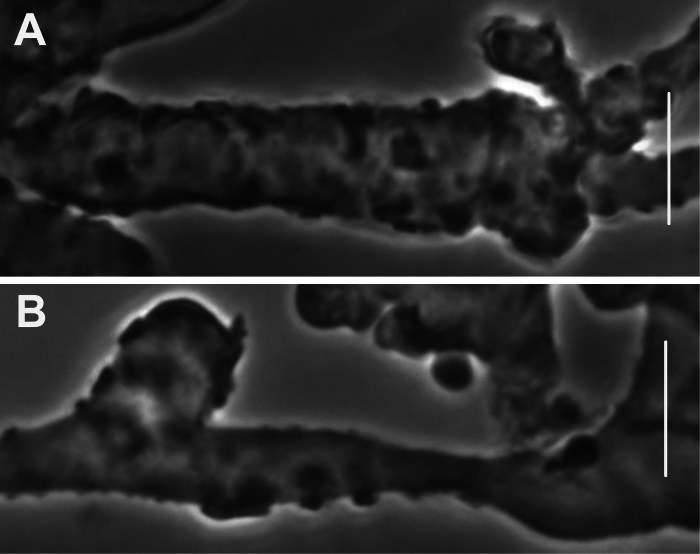
*Collybiopsiscomplicata*. Pileipellis elements; repent, encrusted hyphae. Sx TFB 9168 (TENN-F-055766). Scale bars: 10 mm.

**Figure 5.**
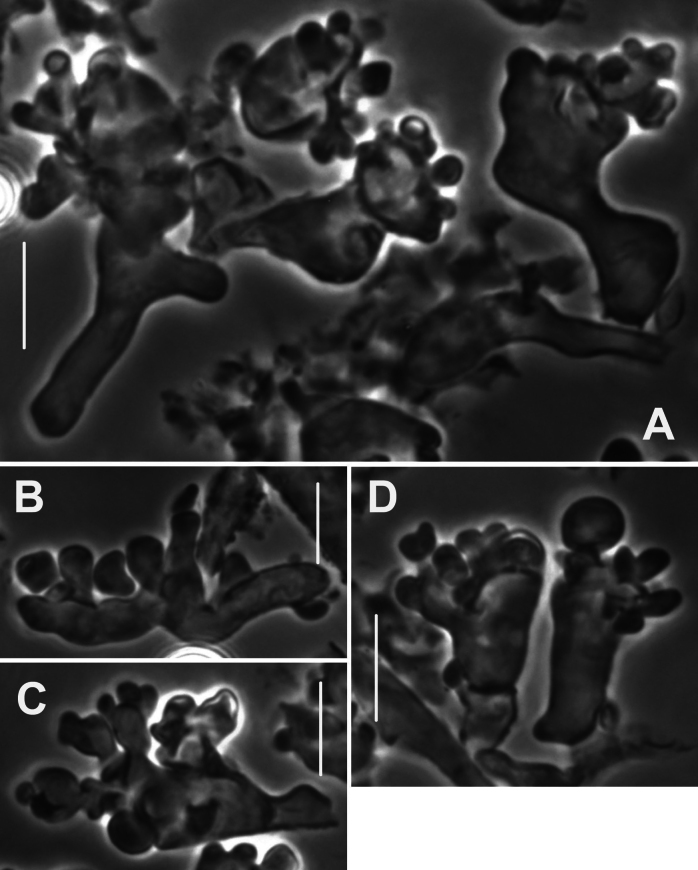
*Collybiopsiscomplicata*. Pileipellis elements; pileocystidia **A** cluster of pileocystidia **B, C** “Diverticulate” repent hyphae **D** two lobose hyphal termini. Note clamp connections. TFB 9168 (TENN-F-055766). Scale bars: 10 μm.

**Figure 6.**
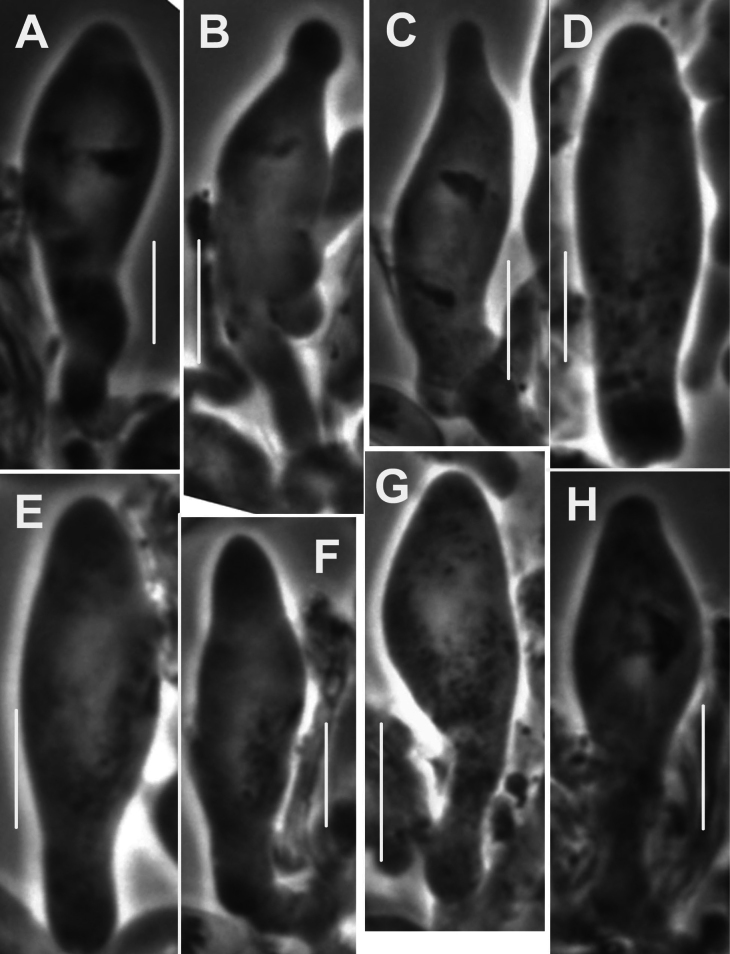
*Collybiopsiscomplicata*. Pleurocystidia Note clamp connection in **C**. TFB 9168 (TENN-F-055766). Scale bars: 10 μm.

**Figure 7.**
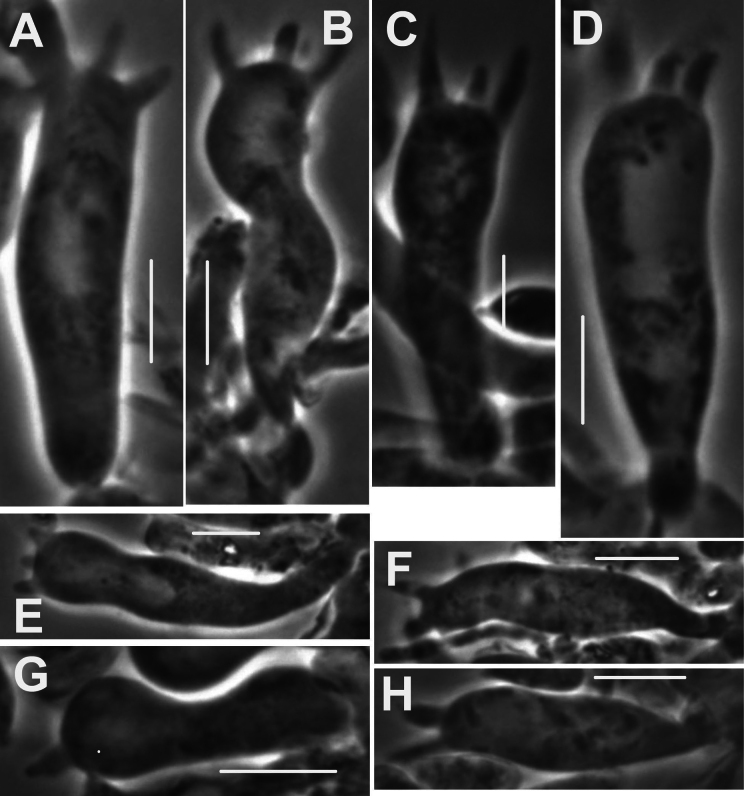
*Collybiopsiscomplicata*. Basidia. TFB 9168 (TENN-F-055766). Scale bars: 10 μm.

**Figure 8.**
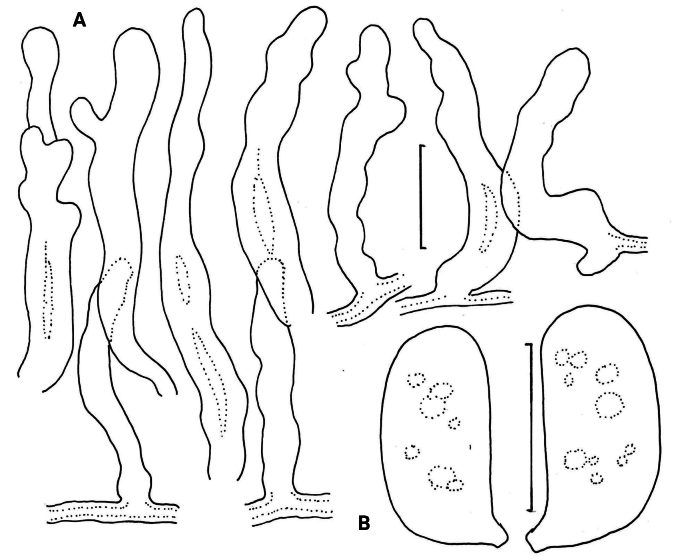
*Collybiopsiscomplicata***A** Caulocystidia **B** Basidiospores. TFB 9168 (TENN-F-055766). Scale bars: 10 μm (**A**); 5 μm (**B**).

**Figure 9.**
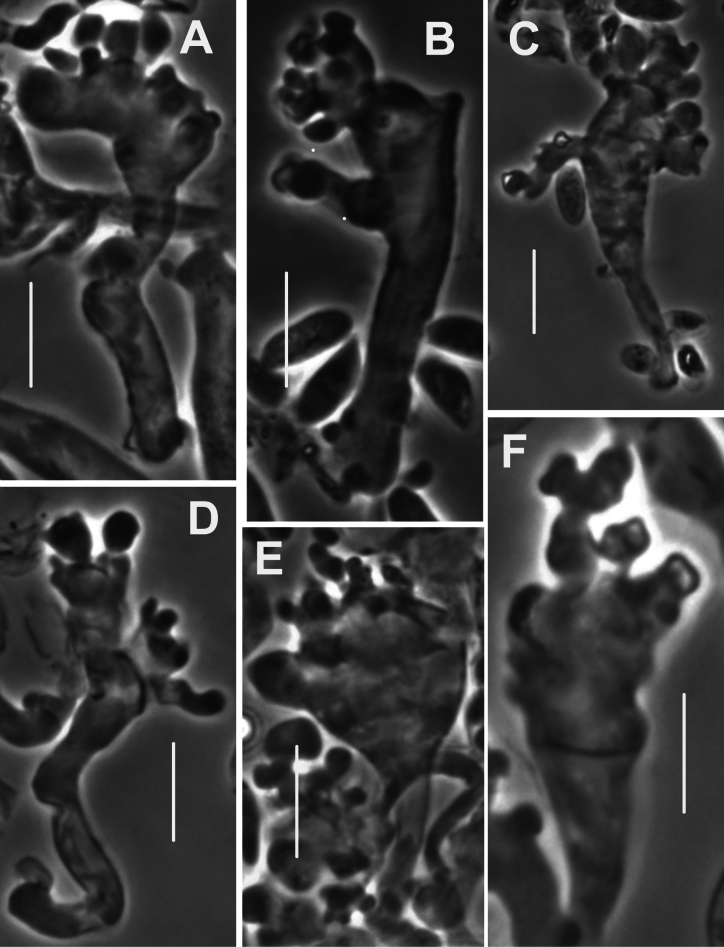
*Collybiopsiscomplicata*. Individual cheilocystidia. TFB 9168 (TENN-F-055766). Scale bars: 10 μm.

**Figure 10.**
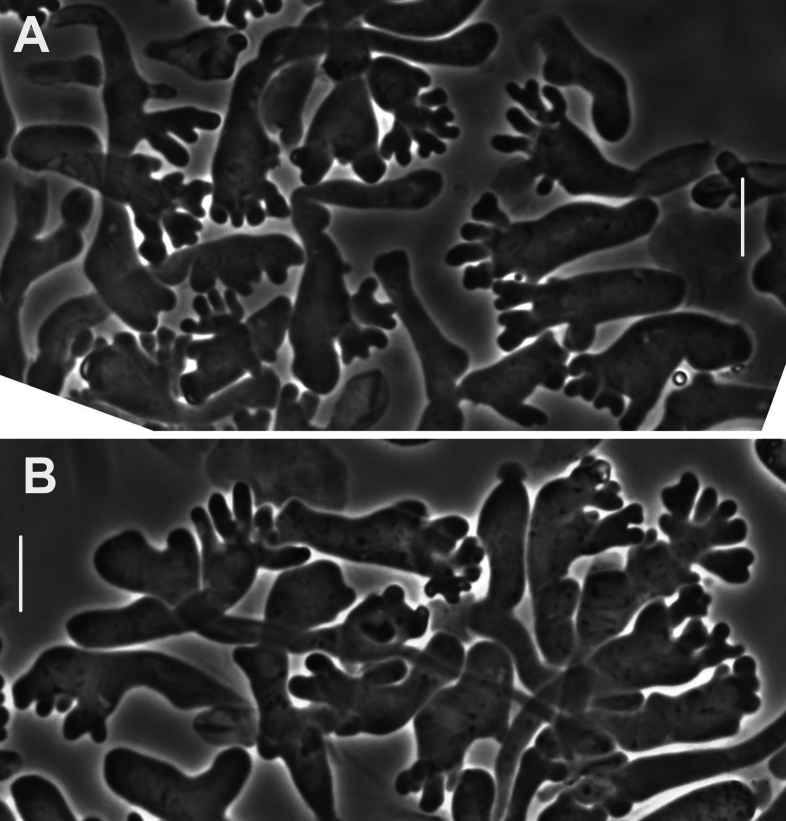
*Collybiopsiscomplicata*. Clusters of cheilocystidia. HUH. HUH-F-00964494. Scale bars: 10 μm.

**Figure 11.**
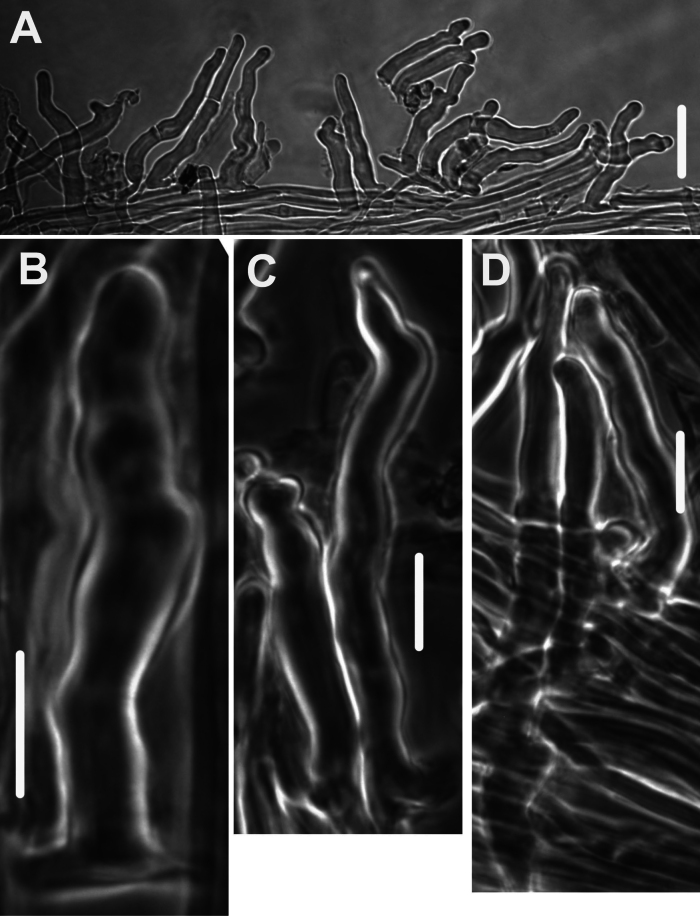
*Collybiopsiscomplicata*. Caulocystidia. TFB 9168 (TENN-F-055766) **A** caulocystidia from upper stipe **B, C, D** individual caulocystidia. Scale bars: 10 μm.

***Pileipellis*** a thatch of occasional repent encrusted hyphae (Fig. 4) and dominant interlocking, stalked-lobose pileocystidia and lobed-diverticulate, repent hyphae (Fig. 5B, C); ***Pileocystidia*** (Figs 5A, D) 27–37 × 5–14 µm, stalked, branching in lobose-coralloid configurations, apparently hyaline, thin- to firm-walled, arising from clamp connections. Subsurface pileipellis hyphae 2.5–10 µm diam, firm-walled, conspicuously clamped, ornamented with small flakes (in profile) but no profile calluses or stripes. ***Pleurocystidia*** (Fig. 6) common, arising from clamp connections, 32–40 × 7–9 μm, stalked-fusiform with apex often broadly submammillate; contents homogeneous. ***Basidioles*** clavate. ***Basidia*** (Fig. 7) 35–60 × 7–9 µm, clavate to weakly urniform or with somewhat expanded subcapitate apex, (2–)4–sterigmate, arising from clamp connections; sterigmata robust, slightly curved; contents heterogeneous, with scattered, refringent granules. ***Basidiospores*** (Fig. 8B) (5.5–)6.5–8.5(–10) × 3–4(–5) µm (Q = 1.56–2.50; Q^m^ = 2.03; L^m^ = 8.02 µm), ellipsoid, flattened adaxially, thin-walled, inamyloid; contents (dried) homogeneous. Lamellar edge sterile. ***Cheilocystidia*** (Figs 9, 10) plentiful, 25–40 × 4–15 µm, stalked, lobose-coralloid branched, often with diverticulate-lobed termini, hyaline, thin-walled. Stipe medullary hyphae 4–14 μm diam, strictly parallel, free (not involved in slime matrix), firm-walled, inconspicuously clamped; contents heterogeneous (multigranular). Stipe cortical hyphae 3–5 µm diam, apparently adherent, obscurely clamped, moderately dextrinoid (more or less hyaline without IKI, reddish brown with IKI), firm- to thick-walled (wall –0.8 μm thick), clamped, producing side branches elongating into caulocystidia. ***Caulocystidia*** from upper stipe (Figs 8 A, 11) in delicate, interrupted patches –80 × 4–10 µm, cylindrical to vermiform, usually somewhat gnarled distally with broadly rounded apex, thick-walled (wall often occluding cell lumen), arising as non-septate side branches from slender (1.5–3.5 µm diam, wall –1.0 µm thick), superficial stipe surface hyphae, strongly dextrinoid (dark brownish red with IKI). Caulocystidia from lower stipe similar, a dense turf of free individuals (not involved in slime matrix), weakly to strongly dextrinoid, –200 × 3–8 µm, thick-walled (wall/lumen distinction often impossible in IKI; wall –1.0 µm thick, less dextrinoid than cell contents), tapering gradually to an acutely rounded apex, often gnarled and sometimes forked. Ample evidence of clamp connections in pileipellis, subpellis and pileus trama (i.e. hook cells of disarticulated clamps common and obvious) occasional complete clamps observed at basidial bases.

##### Habitat.

*Tsuga* debris and adjacent hardwood leaves.

##### Specimens examined.

**Massachusetts**, Plymouth County, Boston Harbor, World’s End Peninsula, coll LA Kappler, 23.VIII.2015, (HUH) BHI 447 (HUH-F-00964493), Boston Harbor, World’s End Peninsula, Rocky Neck, coll D. Healewaters & LA Kappler, 12.VIII.2015, (HUH) BHI 401 (HUH-F-00964494); World’s End Peninsula, coll D. Healewaters et al., 14.IX.2013, (HUH) BHI 034 (HUH-F-00964495. **North Carolina**, Macon Co., vic. Highlands, Bull Pen Rd., Ellicott Rock Trailhead, 35°01.010'N, 83°08.190'W, 20.VII. 2011, coll RHP, TFB 13916 (TENN-F-065811). **Tennessee**, Blount Co., GSMNP, Metcalf’s Bottoms Picnic Area, 9.VI.1997, coll. RHP, TFB 9168 (TENN 55766).

##### Commentary.

The strongly modified “*Rameales*-structure” of the pileipellis structures of *C.complicata* resembles that of species of traditional *Marasmiellus* [viz. *C.ramealis* complex, (Petersen and Hughes 2021)]. This construction comprises a thatch of stalked, ventricose-rostrate structures with lobose, molar-shaped outgrowths. These pileipellis structures conform closely to those of Marasmiellussect.Dealbatisubsect.Dealbatini sensu Singer (Singer 1973) [Type = *Marasmiusdealbatus* Berk. & Curt.], of which *Marasmiusstenophylla* (*C.stenophylla*, Fig. 2) is a member (Desjardin 1997). *M.stenophylla* (section Dealbati) was transferred to *Gymnopus* sensu lato (Mata et al. 2004), then to *Collybiopsis* (Petersen and Hughes 2021). Both Desjardin (Desjardin 1989; 1997) and Hesler (1959) examined type material of *M.subsynodicus* Murrill, considered by Singer (Singer 1973) and Desjardin (Desjardin 1997) to be synonymous with *M.stenophylla* (Mont.) Singer). *Marasmiusdealbatus* remains unplaced in modern classifications.

Parenthetically, in *C.complicata* collections TENN-F-055766 and HUH-F-00964493, the lobose individual pileipellis elements dominated the pileipellis, while in TENN-F-065811 (also *C.complicata*), these structures were only occasional in a pileipellis dominated by encrusted filamentous hyphae.

The pileipellis structure of MarasmiusSect.Androsacei (= Gymnopussect.Androsacei, see Noordeloos and Antonín (2008), is also described as a combination of diverticulate, repent hyphae and *siccus*-type broom cells (a “Rameales-structure”). Our experience with this, however, indicates that the diverticulae (= setulae) of pileipellis cells are much finer, usually vermiform, refringent (PhC) and <1 µm broad. Cheilocystidia of *C.complicata* are very similar to pileipellis structures, but as a rule are somewhat less complexly branched. Morphologically, the pileipellis of *C.complicata* might be forced into section Androsacei, but phylogenetically, it is distant from that group in Gymnopus S.S.

Mata et al. (2007) initially designated TENN-F-055766 (GenBank DQ450029, *C.complicata*) as Marasmiellussp. aff.pluvius, but while basidiomata of *Marasmielluspluvius* Redhead (Redhead 1982) have similar stature, they are smaller and are gregarious on *Pseudotsuga* and *Thuja* needle beds in southern British Columbia. Further, the pileipellis of *Ma.pluvius* does not include stalked-lobose structures, but is a “compactly interwoven layer of densely diverticulate hyphae” (Redhead 1982); “*Rameales*-structure”). Cheilocystidia of *Ma.pluvius* are stalked-vesciculose to clavate with narrow, vermiform setulae quite similar to those of the *Ma.ramealis* complex. Basidiospores of *Ma.pluvius* are longer and significantly narrower than those of *C.complicata* and caulocystidia exhibit a significantly thicker wall than those of *C.complicata*. While perhaps morphologically congeneric with *C.complicata* in *Collybiopsis*, the two species are quite different microscopically and a second GenBank accession under the name Marasmiellusaff.pluvius (MK277736; NL-5034: nrLSU sequence only) is 0.22% different from other *C.complicata* nrLSU sequences. *Marasmielluspluvius* has not yet been transferred to *Collybiopsis*, but DNA sequences will probably support such transfer when they become available, although specific placement in a phylogeny remains unknown.

The nrITS sequences for collections within *C.complicata* (Table 1) are genetically identical with the exception of a 1bp C/T transition in TENN-F-065811 from Macon County, GA (0.17% difference). This lack of nrITS variation includes three nrITS sequences of specimens collected in a study of Boston (MA) Harbor Islands (Haelewaters et al. 2018), and one from the Great Smoky Mountains National Park TENN-F-055766. *Collybiopsiscomplicata*, therefore, seems distributed extensively in temperate Eastern North America. The nearest taxon to *C.complicata*, GenBankaccession MK234195, differs by 6 base pairs (1.05%) and while this falls within the commonly accepted criteria of 2% divergence for conspecificity (Hughes et al. 2009), the lack of variation within *C.complicata* across a wide geographic range (Boston Harbor Islands vs. Southern Appalachians) argues that *C.complicata* does not include MK234195. Possibly, *C.complicata* is a recently-diverging taxon that has not accumulated geographical differences.

#### ﻿ Collybiopsis prolapsis

Taxon classificationFungiAgaricalesOmphalotaceae

95F59265-FFE4-5A1D-A211-1E2063A332E3

Index Fungorum: IF902313

Figs 12 , 13 , 14 , 15 , 16 , 17 , 18 , 19

##### Holotype.

United States, Georgia, Rabun Co., vic. Clayton, Warwoman Dell Picnic Area, 34°52'57.81"N, 83°20'57.99"W, 15.VI.1992, coll. Scott A. Gordon, TFB 4800 (TENN-F-051101).

##### Diagnosis.

1) Basidiomata diminutive, collybioid or marasmielloid, saprophytic on hardwood litter; 2) clamp connections ubiquitous; 3) cheilocystidia “prolapsed,” similar to “ramealis” type, with an abrupt bouquet of branched diverticula; 4) stipe without vesture (i.e. not similar to Gymnopussect.Vestipedes; 5) resupinate patch significant, with diminutive, white hyphal ropes; 6) necropigment weak over hymenophore; 7) nrITS sequence unique, but quite similar to that of *C.complicata* and *C.minor*.

##### Etymology.

Pileo- and cheilocystidia structures with swollen, subspherical excrescences, reminiscent of a prolapse.

##### Description.

**Figure 12.**
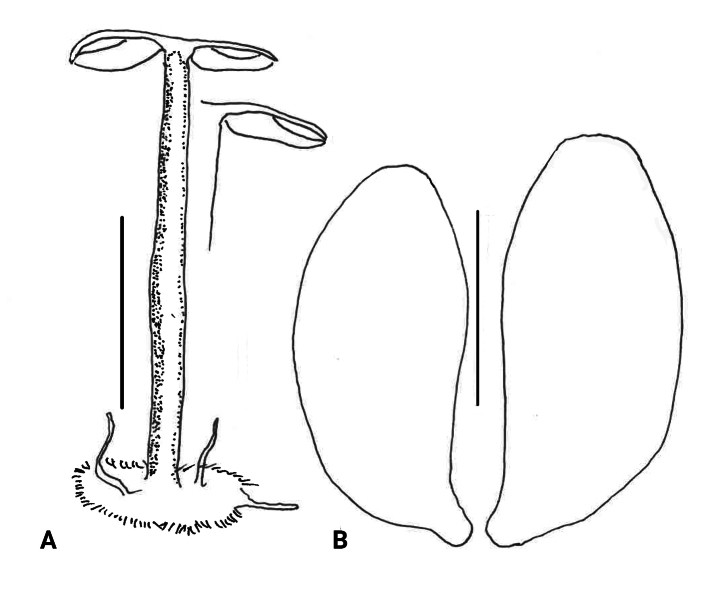
*Collybiopsisprolapsis***A** basidioma **B** basidiospores, Scale bars: 8 mm (**A**); 5 µM (**B**).

Basidiomata diminutive (Fig. 12). ***Pileus*** 8–12 mm broad, when fresh rich deep brown (6E6-7, “Brussels brown,” “Sudan brown”), shallowly convex to plane, very vaguely finely sulcate, minutely radially fibrillose; drying grayish brown, more or less unicolorous. ***Lamellae*** subdistant, adnate (with very slight non-lamellate hymenium decurrent on stipe apex for less than a millimeter), not ventricose (straight from stipe to margin), total lamellae 30–40, through lamellae 4–10, “off-white” to “cream” to “brown orange”, 6C4 in age; lamellulae rudimentary. ***Stipe*** when fresh reported as concolorous with pileus, when dried more or less concolorous with lamellae, terete, hardly twisted, glabrous-shining to somewhat wispy very near base; stipe base with extensive (–1.5 sq cm) resupinate patch, now creamy off-white and appearing varnished and with a few small, off-white synnematoid mycelial ropes. ***Odor*** and ***taste*** not recorded.

**Figure 13.**
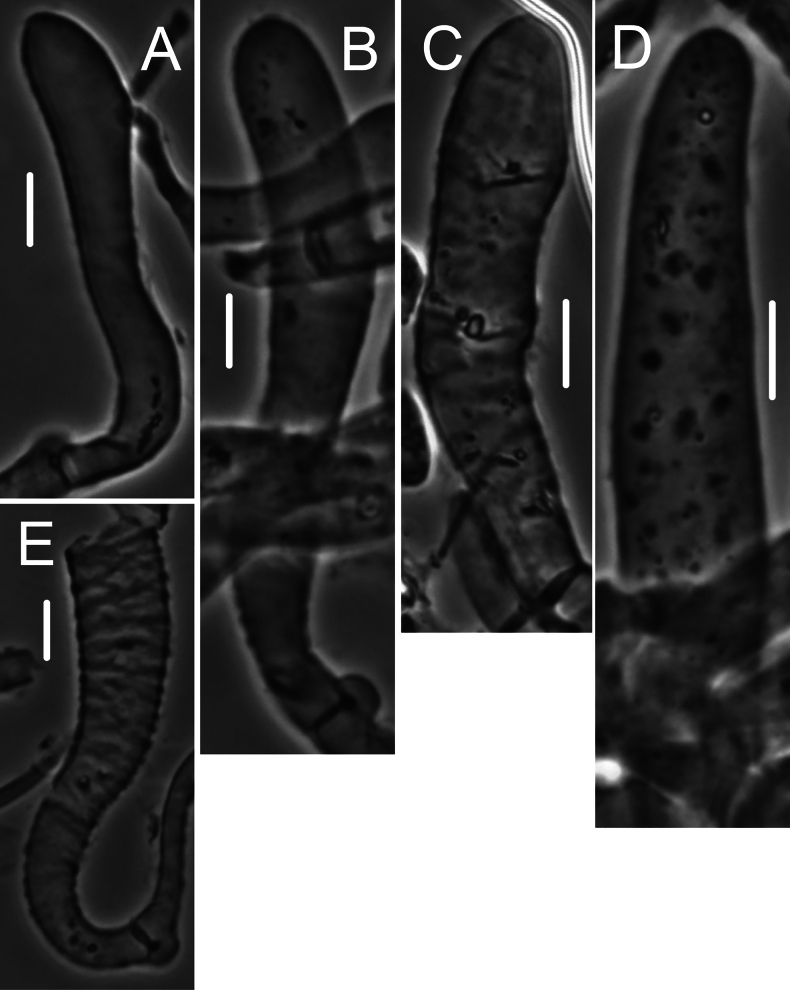
*Collybiopsisprolapsis*. Dermatocystidia. Scale bars: 10 µM.

**Figure 14.**
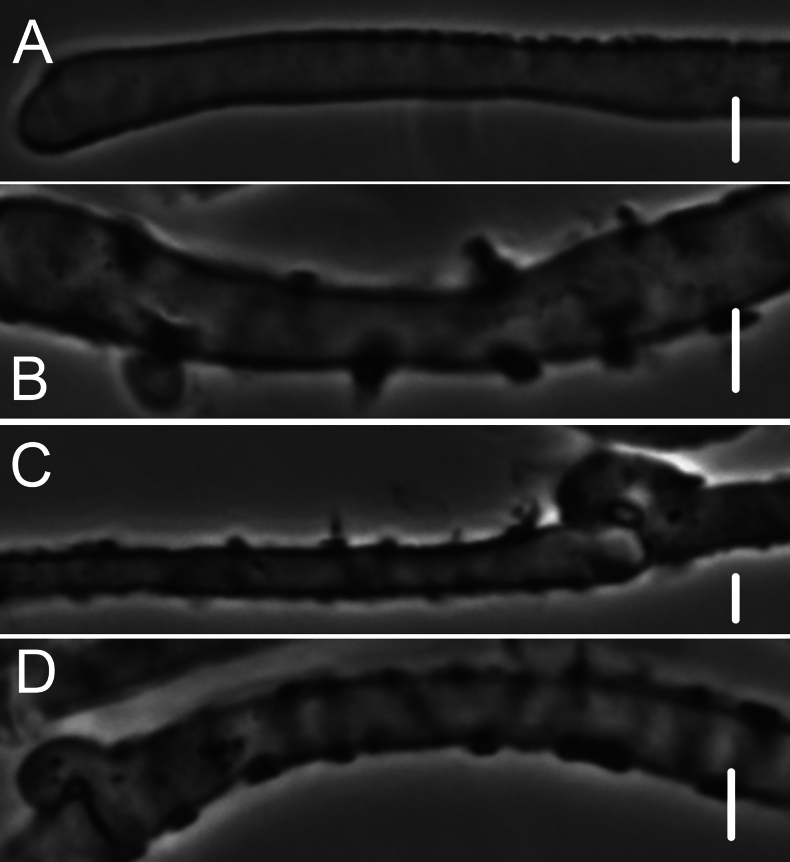
*Collybiopsisprolapsis*. Repent pileipellis hyphae. Scale bars: 5 µM.

**Figure 15.**
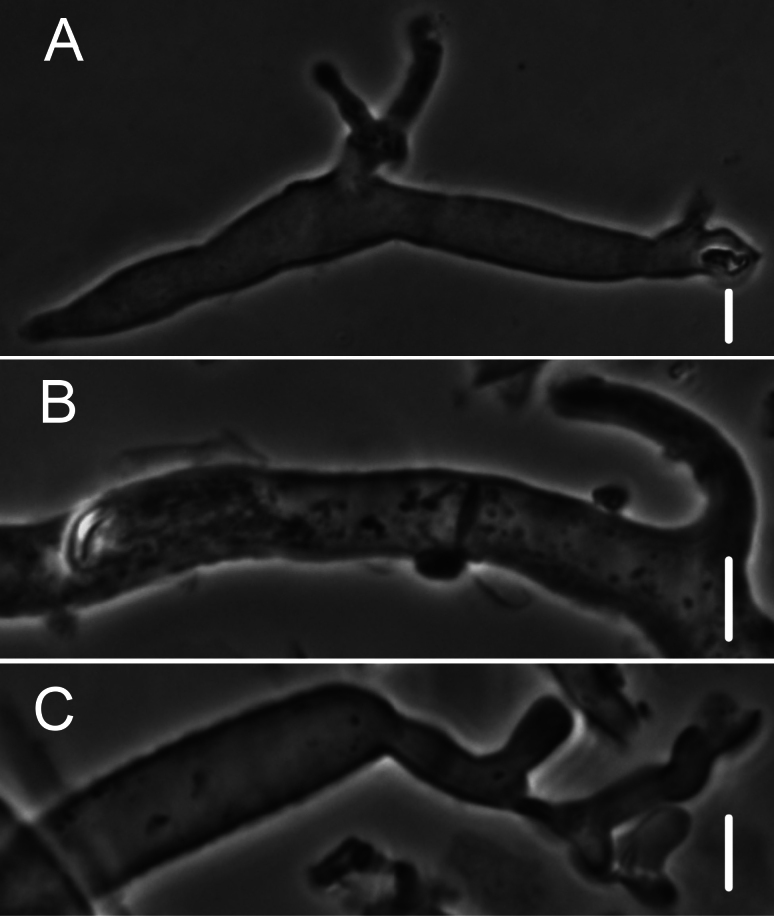
*Collybiopsisprolapsis*. Smooth, repent hyphae with ‘Subfumosae’ side branches. Scale bars: 5 µM.

**Figure 16.**
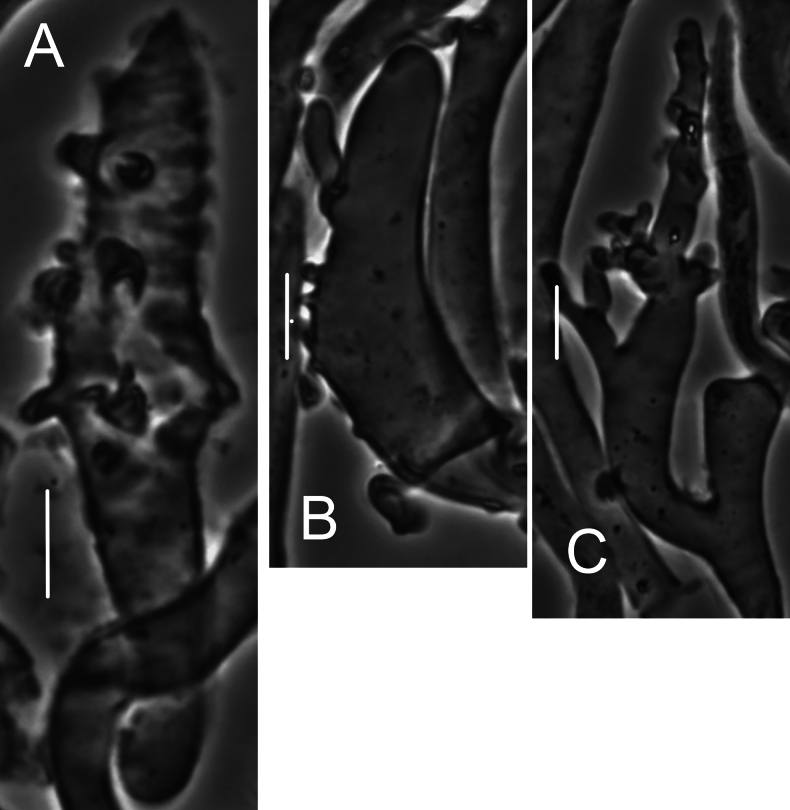
*Collybiopsisprolapsis*. Dermatocystidia, thick-walled, inflated, often ornamented. Scale bars: 7.5 µM.

**Figure 17.**
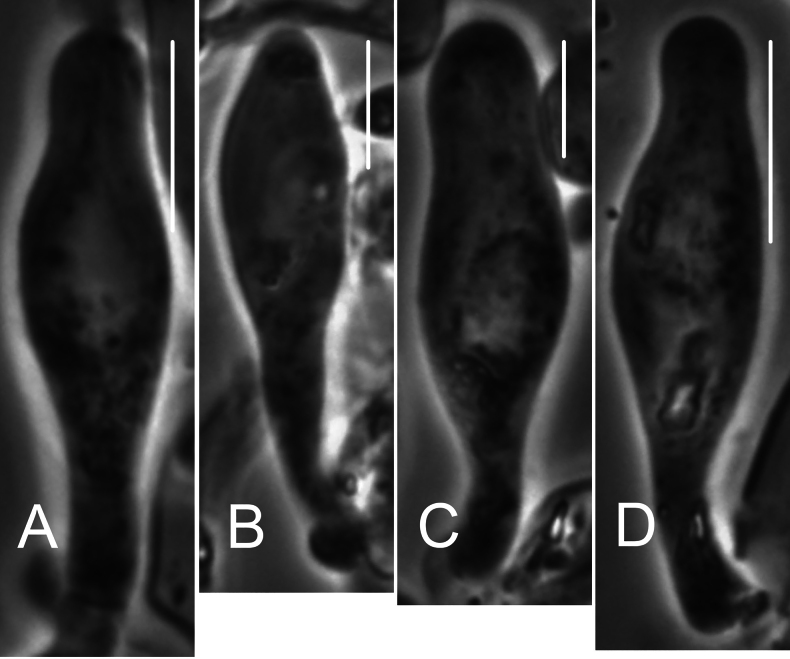
*Collybiopsisprolapsis*. Pleurocystidia. Scale bars: 30 uM.

**Figure 18.**
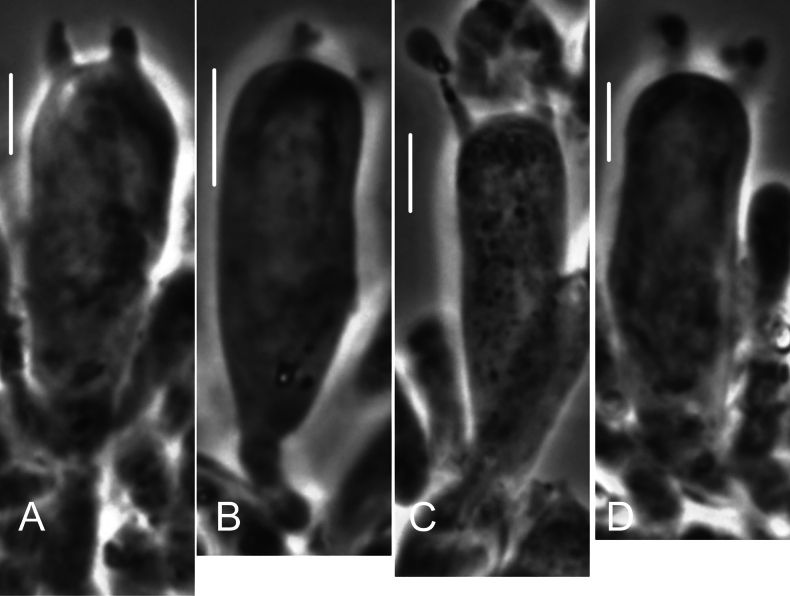
*Collybiopsisprolapsis*. Basidia. Scale bars: 30 uM.

**Figure 19.**
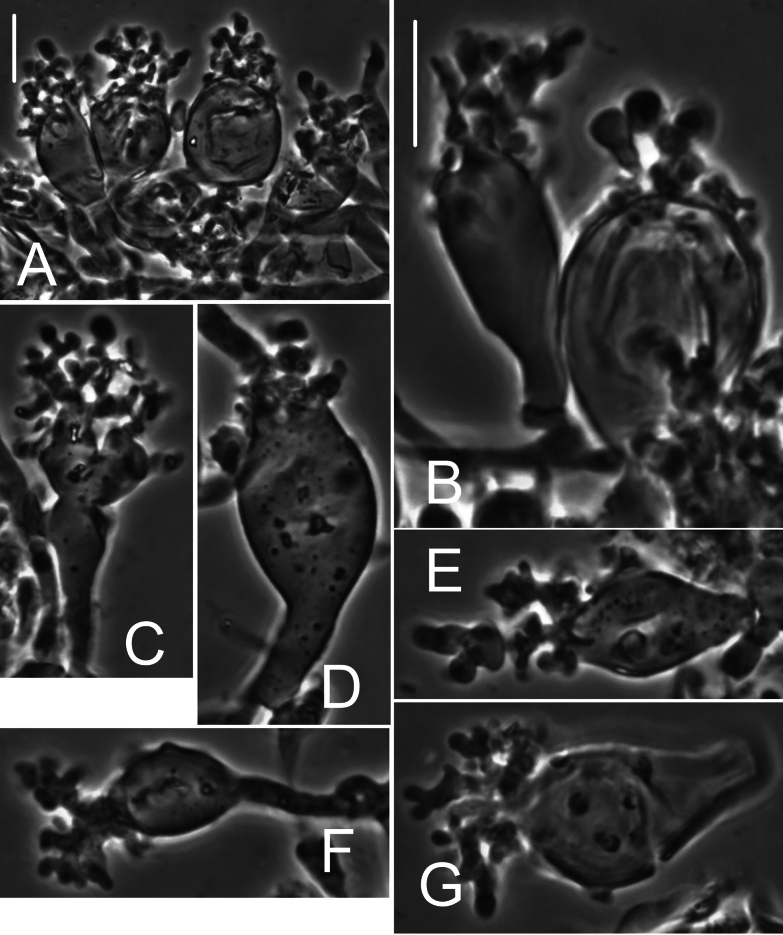
*Collybiopsisprolapsis*. Cheilocystidia. Clusters with diverticula (**A, B)** Individual Cheilocystidia (**C–G)**.Scale bars: 16 uM (**A, B**).

***Pileipellis*** a repent layer of free (with no evidence of slime matrix or individual slime sheath), filamentous hyphae of the following types: 1) dermatocystidia (Fig. 13) clavate to fusiform to inflated and tapering distally, 7–11.5 μm diam, smooth (Fig. 13A, B) to ornamented in annular pattern (Fig. 13C, E), apparently arising at a clamp connection; contents homogeneous to heterogeneous with scattered inclusion (Fig. 13D); 2) repent hyphae 3.5–8 μm diam, thin- to firm-walled, varying as follows: a) minutely roughened (Fig. 14A); b) ornamented with individual scabs, flattened in flake-like scales, individual lumps –2 μm high (Fig. 14B), spiculate structures (Fig. 14C) and/or annular ornamentation with profile calluses (Fig. 14D); 3) smooth, repent hyphae 3.5–7.5 μm diam, with occasional filamentous side branches (Fig. 15A–C); branches simple, lobate to branched, not arising from clamp connections; and 4) thick-walled, usually somewhat inflated hyphae 5.5–10.5 μm diam, with thick-walled cog-like warts, 1.5–2.5 × 1–2.5 μm (Fig. 16A, B); intermediate forms (Fig. 16C) occasional. Pileal and lamellar tramae interwoven; hyphae 3–8.5 μm diam, firm-walled, frequently and conspicuously clamped, in lamellar trama with slender hyphae 2–3.5 μm diam. ***Pleurocystidia*** (Fig. 17) 24–34 × 7–11 μm, abundant, stalked-fusiform, near lamellar edge ampulliform with rounded apex, conspicuously clamped; contents homogeneous to heterogeneous with crystal-like inclusions. ***Basidia*** (Fig. 18) more or less stalked-columnar (not urniform, not clavate), 27–35 × 8–12 μm, 4-sterigmate (sterigmata slender, slightly curved), conspicuously clamped; contents homogeneous to minutely heterogeneous. ***Basidiospores*** (Fig. 12B) (9–)9.5–10.5 × 4–4.5(–5) μm (Q = 2.10–2.50; Q^m^ = 2.05; L^m^ = 9.75 μm), elongate-ellipsoid, somewhat tapered proximally, thin-walled, hyaline; contents homogeneous to heterogeneous-subrefringent. ***Cheilocystidia*** (Fig. 19 A–G) ventricose-rostrate to stalked-globose, hyaline, firm- to thick-walled (wall –1 μm thick, especially laterally, smooth), conspicuously clamped, apically producing a cluster of diverticula; diverticula –16 × 1–2.5(–4) μm, repeatedly dichotomously branched, often inflated somewhat apically (Fig. 19 A, B). ***Stipe medullary hyphae*** thick-walled, occasionally conspicuously clamped, of two types: 1) 4.5–8.5 μm diam, seldom branched; contents heterogeneous (multigranular); and, 2) 2–3.5 μm diam, occasionally branched; contents homogeneous. Stipe cortical hyphae similar to slender medullary hyphae. ***Resupinate*** patch composed of tightly interwoven hyphae in a slime matrix; hyphae of two types, both inconspicuously clamped: 1) 3–5.5 μm diam, thick-walled (wall –1 μm thick, refringent; PhC); and 2) 2.5–4 μm diam, thick-walled (wall –0.7 μm thick, non-refringent), frequently branched.

##### Commentary.

Specimen notes on undried specimens for the holotype specimen, TENN-F-051101, report lamellae as brownish-orange (6C4) in age. Similar pigmentation is currently evident on dried material, presumably a necropigment (approximately “Light ochraceous salmon,” “Light salmon orange,” more or less characteristic of the *Collybiopsisramealis* complex.)

The pileipellis is a poorly developed *Gymnopus* structure (Hughes and Petersen 2015), with only a few “diverticulate” hyphal termini as described by Halling (1983); typical of section Subfumosae. Conversely, the typical *Ramealis* pileipellis structure is quite different. There, the typical diverticula are cog-like (not wart-like) and the surrounding hyphal walls thickened (vaguely similar to those shown in Fig. 16A, B). Clamp connections are common and conspicuous throughout. The thick walls of stipe medullary hyphae appears to be laminate, often peeling into narrow shards (as in peeling a banana).

Cheilocystidia, while highly distinctive, are not totally unique. *Collybiopsisstraminipes* cheilocystidia are similar, but the specimens examined (Desjardin and Petersen 1989, including the type) were clampless and from spruce-fir zone. A clamped variety (Marasmiusstraminipesvar.fibulatus Desjardin & R.H. Petersen) is from lower elevation and hardwood (*Quercus* litter) substrate.

## ﻿Discussion

The fragmentation and rearrangement of the agaricoid Omphalotaceae, *Marasmius* and *Marasmiellus* is ongoing as molecular data identifies new taxa and associations (Owings and Desjardin 1997; Wilson and Desjardin 2005; Matheny et al. 2006; Mata et al. 2007; Hughes and Petersen 2015; Petersen and Hughes 2016; Petersen and Hughes 2017; Petersen and Hughes 2021). This understudied group of small gymnopoid mushrooms will continue to enlarge as environmental studies identify new members and define their niches.

## Supplementary Material

XML Treatment for Collybiopsis complicata

XML Treatment for Collybiopsis prolapsis

## ﻿Additional information

### Author ORCIDs

Ronald H. Petersen https://orcid.org/0000-0002-3170-5770

Karen W. Hughes https://orcid.org/0000-0003-2865-8798

### Data availability

All of the data that support the findings of this study are available in the main text.
